# Novaferon, a novel recombinant protein produced by DNA-shuffling of IFN-α, shows antitumor effect *in vitro* and *in vivo*

**DOI:** 10.1186/1475-2867-14-8

**Published:** 2014-01-27

**Authors:** Meng Li, Chunming Rao, Dening Pei, Lan Wang, Yonghong Li, Kai Gao, Minrong Wang, Junzhi Wang

**Affiliations:** 1 National Institutes for Food and Drug Control of China, No. 2 Tiantan Xili, Beijing 100050, China; 2 Beijing Genova Biotech Company, Beijing 100050, China

**Keywords:** Novaferon, Recombinant interferon-α protein, Anti-cancer, *in vivo*, IFN receptor 2

## Abstract

**Objective:**

A recombinant antitumor/antiviral protein (Novaferon, Nova) is a new type of interferon, which is produced by artificial design technology combining DNA-shuffling and High Throughput Screening (HTS).

**Methods:**

The *in vitro* biological activities, such as anti-tumor activity and antiviral activity of Nova and recombinant human interferon alpha-2b (rhIFN-α2b) was performed; *in vivo* anti-tumor activity in nude mice was also tested. Flow cytometry, histo-pathological analysis including HE staining and immunohistochemistry, and surface plasmon resonance assay were performed to investigate the underlying mechanisms analysis.

**Results:**

Nova exhibited stronger anti-cancer effects compared to rhIFN-α2b i*n vitro* and *in vivo*. The antitumor mechanisms of Nova may be related to S phase arrest, pro-apoptosis, and inhibition of tumor angiogenesis. Moreover, Nova exhibited a higher binding affinity for IFN receptor 2 (IFNR2) than rhIFN-α2b, which is one of the possible reasons accounting for its stronger actions against tumor cells compared with rhIFN-α2b.

**Conclusion:**

Nova has strong antitumor activity and could be a potentially effective therapeutic drug for cancer.

## Introduction

Cancers are becoming a leading cause of morbidity and mortality worldwide in the coming decades. At present, the treatment of cancer is the major challenge for the global medical community [1]. With the advance of molecular biology and genetic engineering, the development of anticancer drugs has entered a new stage. Considerable progress has been made in harnessing biotechnology for the development of anticancer drugs, such as monoclonal antibodies, cytokines and interferons.

Interferon (IFN) is one of the most common biotechnology drugs which has been widely used in the adjuvant therapy of tumors [2,3]. Clinical benefits of IFN-α therapy includes increase in the disease-free survival by ~9 months and the 5-year survival by 8–9% [4,5]. Interferon possesses both direct tumor-killing and immunomodulatory effect [6], IFN-α exerts its effects mainly through the Tyk2/Jak1-STAT1/STAT2 pathway to activate transcription from the IFN-stimulated response element (ISRE) [5]. IFN-α is currently the most used cytokine in the treatment of cancer. However, the potential anti-tumour activity of IFN-α is limited by the activation of tumour resistance mechanisms [7].

Novaferon (Nova) is a new recombinant IFNα like anti-tumor/viral protein with significantly higher activities than IFN-α [8]. Screened from over 10 million artificial clones of recombinant interferons. Preliminary analysis suggest it may have more strong bioactivity than normal IFNα and share the same mechanism on cancer treatment with interferon in cell cycle arrest, promotion of apoptosis and inhibition of oncogene expression.

In the current study, we tested Nova’s bioactivities on tumor cells and virus; secondly, we also probed the mechanisms of Nova in cancer treatment.

## Materials and methods

### Animals and cell lines

Male BALB/c nude mice, 6 weeks old, were purchased from the National Institutes for Food and Drug Control. The mice were fed under pathogen-free and humane conditions. All experiments involving the use of mice were carried out according to the protocols approved by the Animal Care and Use Committee of Academy of Military medical Sciences (Beijing, China). WISH cells (CCL-25), HepG2 (HB-8065) and Daudi cells (CCL-213) were purchased from ATCC (Manassas, VA, USA). HepG2 cells were cultured in Eagle's Minimum Essential (EME) medium (Gibco, NY, USA) supplemented with 10% (v/v) FBS (Invitrogen, CA, USA) in a humidified atmosphere containing 5% CO2 at 37°C. WISH cells were cultured in Minimal Essential Medium (MEM) supplemented with non-essential amino acids and 10% (v/v) FBS. Daudi cells were cultured in RPMI-1640 medium (Gibco, NY, USA) supplemented with 10% (v/v) FBS (Invitrogen) in a humidified atmosphere containing 5% CO2 at 37°C.

### Reagents

Nova was prepared according to method described previously [9], in brief, a shuffling library with 12 subtypes of IFNαs was constructed from human leukocyte cDNAs. Followed by PCR amplification, DNase I digestion and anti-tumor/virus activity screening, Novaferon, a human interferon-like protein was screened. The purity of Nova was determined by Size Exclusion Chromatography-High- Performance Liquid Chromatography (SEC-HPLC) assay. Nova was separated by Shodex Protein KW-803 column (Showa Denko America, Inc., NY, USA) in PB buffer (20 mM Na3PO4, 0.5 mM NaCl, 0.02% Tween80, pH7.0). The purity was calculated by division of the target peak area and the total peak area.

Antibody against caspase-3 cleaved fragment was purchased from Upstate Biotechnology (Temecula, CA, USA). Antibodies against Bax and Bcl-2 were purchased from Santa Cruz Biotechnology (Santa Cruz, CA, USA). Antibody against Ki-67 was purchased from Thermo Fisher Scientific (Melbourne, VIC, Australia). Antibody against α-SMA was purchased from Abcam (Cambridge, MA, USA). Recombinant human IFN-α/β R2-Fc chimera, recombinant human IgG1 Fc and goat anti-human IgG Fc was purchased from R&D Systems (Minneapolis, MN, USA). FITC Annexin V/PI apoptosis kit was from BD Biosciences (Burlington, MA, USA).

### Antiproliferative test in Daudi cells

For the MTT [3-(4,5-dimethylthiazol-2-yl)-2,5-diphenyltetrazolium bromide; Sigma-Aldrich, Inc., St. Louis, MO] anti-proliferative assay, Daudi cells (3 × 10^4^ cells/well) were treated with Nova, rhIFN-α2b and IFN-α international standard control at an initial concentration with 4-fold serial dilutions (0 ~ 250 IU/ml) in 10% FBS-RPMI as previously described [10]. Concentrations of IFNs that inhibit cell growth by 50% (IC_50_) were calculated at 48 h after treatment.

### Anti-viral activity testing by WISH/VSV assay

Vesicular stomatitis virus (VSV) has been reported to induce apoptosis and the onset of apoptosis may play an important role in virus-associated diseases [11,12]. We compared anti- viral activity of Nova and rhIFN-α2b by virtue of cytopathic effect reduction assay (CPE) that employed a human amnion cell line(WISH) challenged with vesicular stomatitis virus (VSV) as described in detail elsewhere [13], inoculated WISH cells were added in a 96-well plate at a density of 3 × 10^5^ cells/ml, subsequently treated with 4-fold serial dilutions (0 ~ 250 IU/ml) of Nova, rhIFN-α2b and IFN-α international standard control for 24 h. A total of 1000 pfu VSV was added to infect cells for 12 h. The staining and dissolve procedures of WISH cell/VSV system were described previously [14]. Anti-tumor/viral activity of Nova and rhIFN-α2b was demonstrated as absorption value data of 550 nm after analyzed by Soft max program.

### Anti-tumor activity tested by flow cytometry

A total of 1 ml HepG2 cells (10^5^/ml) were added into a 12-well plate, cells were treated with Nova and rhIFN-α2b, untreated cells served as blank control. Twenty four hours later, the cells were stained by Propidium Iodide (PI) [15] and harvested. Stained nuclei were analyzed using BD FACScan flow cytometer (Becton Dickinson, NY, USA). DNA distributions were analyzed by BD FACSDiva software (BD Biosciences, NJ, USA) for the proportions of cells in S phase of the cell cycle.

### In vivo anti-tumor activity in nude mice

Tumors were implanted in BALB/c-nude mice (20–25 g; n = 32) as previously described [6]. Briefly, 100 μl of HepG2 cells (1 × 10^6^, viability of 95–97%) were inoculated into the shoulder of right forelimb. The mice were housed one per cage with free access to sterile water and standard laboratory chow diet. When the diameter of the tumors reached to 4-5 mm, the mice were randomly divided into four groups (N = 6): Nova group, rhIFN-α2b group, positive control group (fluorouracil) and negative control group (PBS). Subcutaneous injection was carried out with varied doses in different groups: Nova 250 μg/kg, rhIFN-α2b 250 μg/kg, fluorouracil 30 mg/kg, PBS 0.1 ml. Nova, rhIFN-α2b and PBS were administrated once daily for 21 days. Fluorouracil was administrated every other day. Twenty one days later, anesthetization of sodium pentobarbital was applied to the mice and then the mice were sacrificed; RPR (relative proliferation rates) with the tumor volumes and the TIR (tumor growth inhibition rates) with the tumor weights were calculated respectively based on previously similar study [16].

### HE and Immunohistochemistry analysis

Excised tumor specimens were fixed in 10% neutral-buffered formalin. After embeded in paraffin, a series of 5-μm sections were sliced. Sections were de-paraffinized, and rehydrated. After the sections were de-waxed by xylene and processed by graded ethanol debenzolization. They were then washed with tap water, differentiated with hydrochloric acid and ethanol before staining with eosin for 2 min. For Immunohistochemistry analysis, after quenching endogenous peroxidase activity and blocking nonspecific binding sites, slides were incubated at 4°C overnight with 1:100 dilutions of primary antibodies against Bax, Bcl-2, caspase 3, Ki-67 and α-SMA respectively, followed by 30-min incubation with secondary antibodies. Slides were then visualized using the DAB chromogen (Lab Vision Corp., Fremont, CA).

### Surface Plasmon Resonance (SPR) assay

SPR method is one of effective methods to study direct macromolecular interactions, compared with indirect methods, such as ELISA; SPR has the real-time, rapid, and label-free characteristics. For analysis of Nova and IFN-a2b affinity for IgG, SPR studies were performed using a BIAcore T-100 (BIAcore AB, Uppsala, Sweden). Human IFNR2-Fc was captured on the CM5 chip by an immobilized anti-human IgG Fc antibody. Nova or IFN-α2b was added in the solution phase, the binding was measured under equilibrium conditions. The data were analyzed by BiacoreT-100 evaluation.

## Results

### Sequence alignment and purity of nova

**Figure 1:**
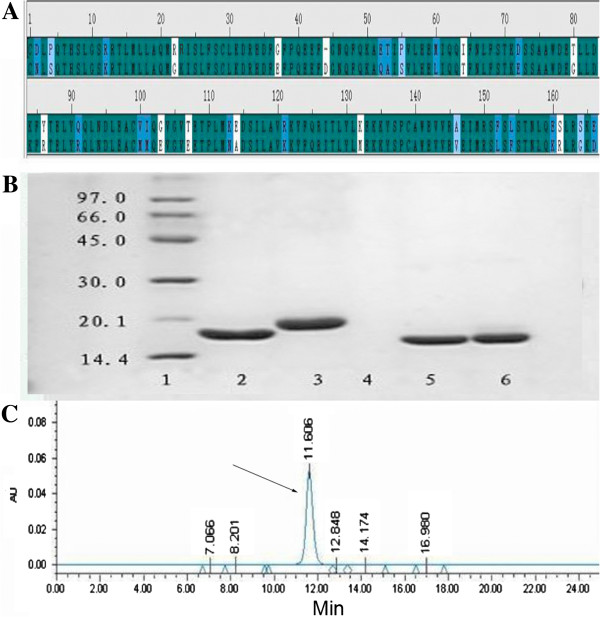
**Sequence, molecular weight and purity of Nova. (A)** Sequence alignment between IFN-α2b (upper line) and Nova (lower line); **(B)** SDS-PAGE separation result (stained with Commassie Brilliant Blue R-250): Lane 1, marker; Lane 2, rhIFN-α2b (Reduction), 17.51 KD; Lane 3, Nova (reduction), 19.06 KD; Lane 4, Blank; Lane 5: rhIFN-α2b (Non-reducing, 100%); Lane 6, Nova (Non-reducing, 100%); **(C)** Size-exclusion high performance liquid chromatography analysis result, arrow indicated the Nova.

Nova shares approximately 82% sequence identity to human IFN-α2b (Figure 1A). The molecular weight of Nova was about 19 kDa estimated by SDS-PAGE (Figure 1B). The purity of Nova was at least 99% tested by SEC-HPLC (Figure 1C).

### Anti-tumor/virus activity of nova

**Figure 2:**
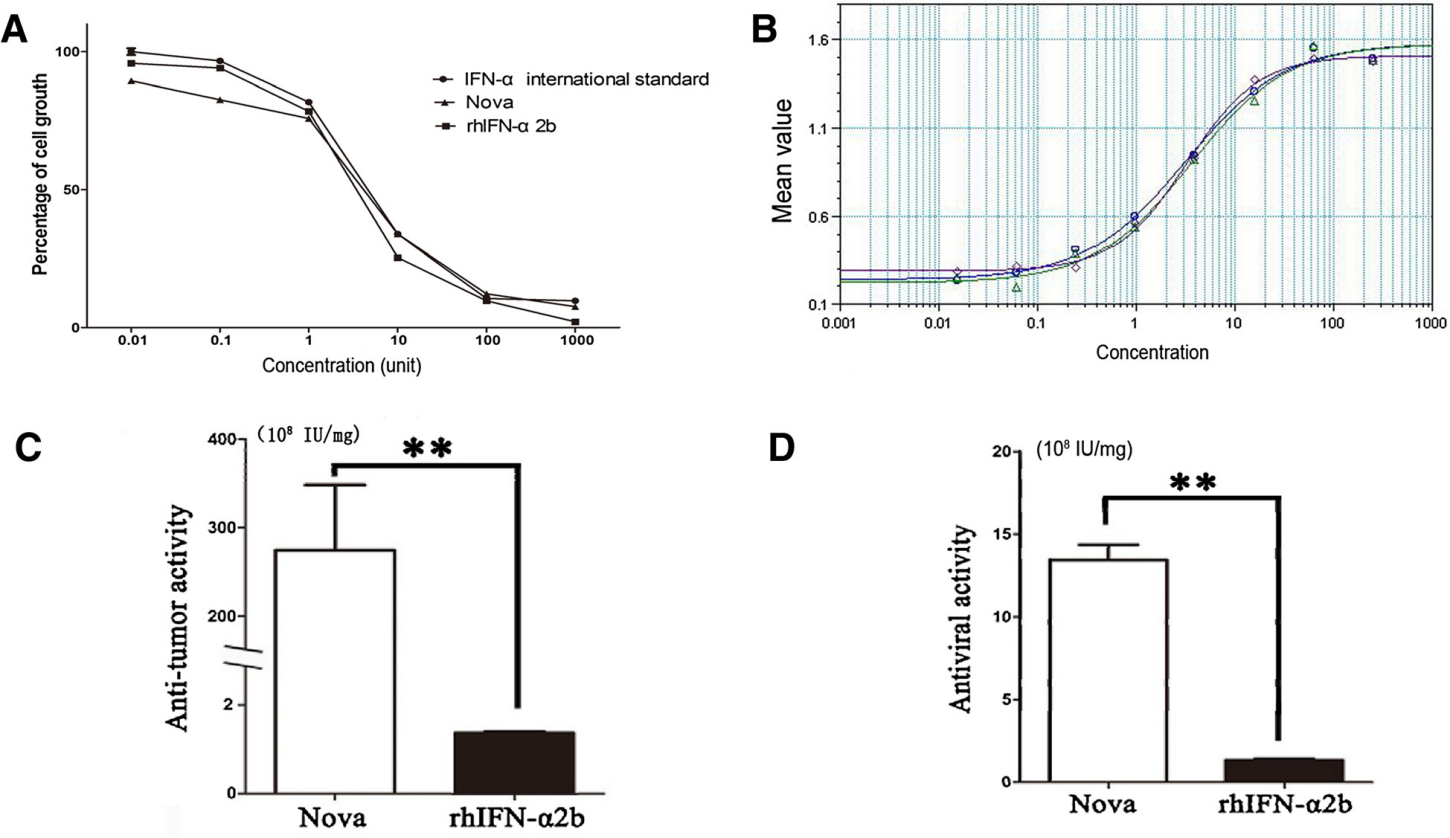
**The Comparison of Nova and rhIFN-α2b in anti-virus/tumor activity.** The anti-tumor **(A)** and anti-virus **(B)** activity of Nova and rhIFN-α2b determined by WISH-VSV system and Daudi-MTT assay, ◇:rhIFN-α2b, Δ: Nova, Ο: IFN-α international standard control; The column demonstration of anti-tumor **(C)** and anti-virus activity **(D)** of Nova and IFN-α2b, ^**^, P < 0.01, compared with IFN-α2b.

The anti-tumor activity of Nova and IFN-α2b against Daudi cell lines was 2.74 × 10^10^ U/mg ± 8.71 × 10^9^ U/mg (n = 3) and 1.57 × 10^8^ U/mg ± 6.91 × 10^7^ U/mg (n = 3) respectively (Figure 2A). The anti-virus activity of Nova and IFN-α2b, which was verified by WISH/VSV system, was 1.34 × 10^9^ ± 1.57 × 10^8^ (n = 3) and 1.34 × 10^8^ ± 1.48 × 107 (n = 3) respectively (Figure 2B). Nova shared a high homology with IFN-α2b, but the anti-tumor and anti-virus activity of Nova was 174-fold (Figure 2C) and 10-fold (Figure 2D) greater than IFN-α2b.

### Enhanced inhibition of nova to Hep-G2 cell proliferation in nude mice

**Figure 3:**
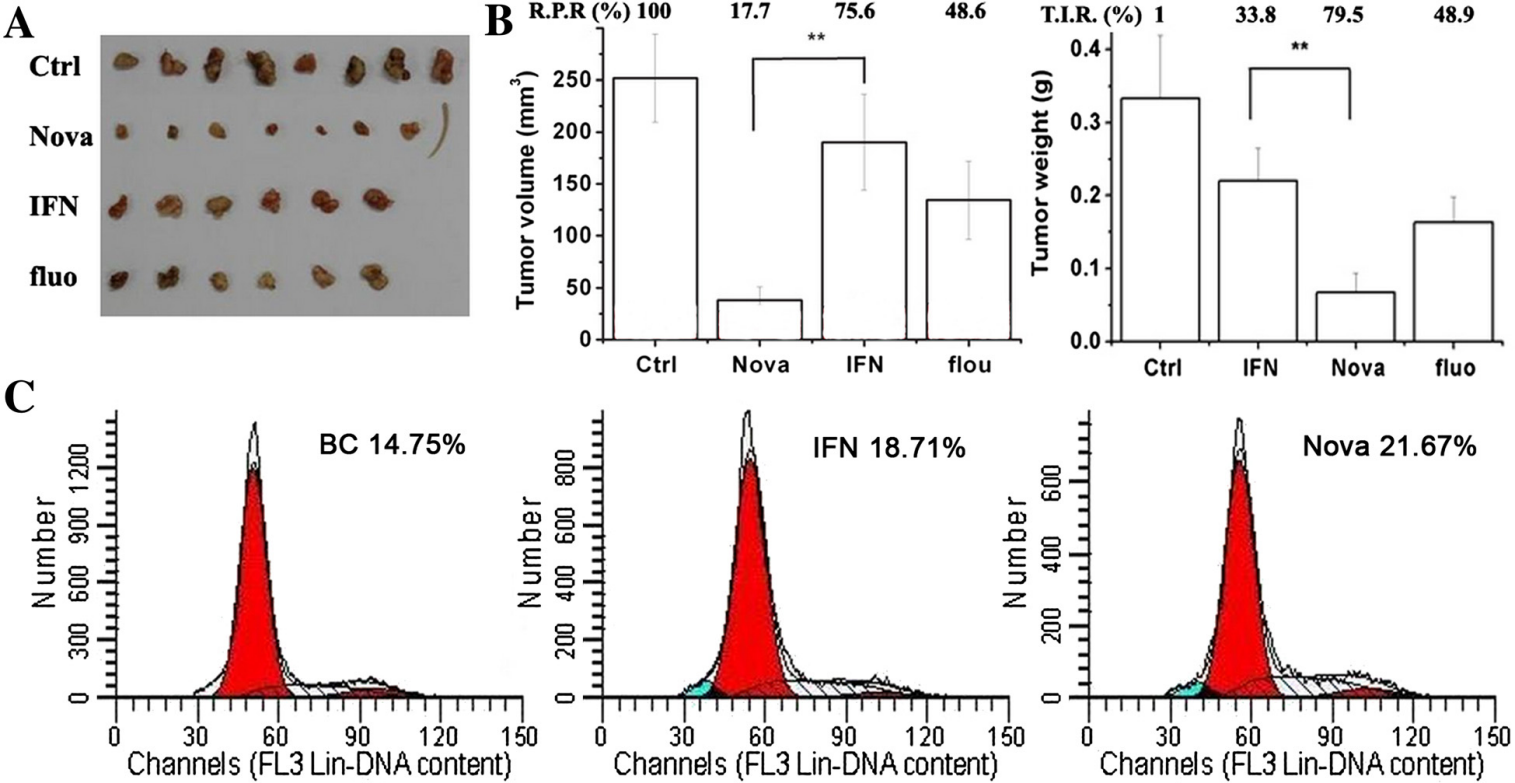
**In vivo anti-proliferative activity of Nova. (A)** HepG2 tumor volume in each group; (**B**, left) The relative proliferation rates (R.P.R.) of Nova, IFN-α2b and fluorouraci. (**B**, right) The tumor growth inhibition rates (T.I.R.) of Nova, IFN-α2b and fluorouracil. **(C)** The cell cycle through propidium iodide (PI) staining, specific S phase cell cycle inhibition was observed in Nova and IFN-α2b groups.

To explore the anti-proliferative activity of Nova *in vivo*, effect of Nova on Hep-G2 cell proliferation in nude mice was tested. Result showed IFN-α2b and fluorouracil reduced the HepG2 tumor volume (Figure 3A), and was consistent with previous reports [17,18]. The relative proliferation rates (R.P.R.) of Nova, IFN-α2b and fluorouracil were 17.7%, 75.6% and 48.6%, respectively (Figure 3B, left). The tumor growth inhibition rates (T.I.R.) of Nova, IFN-α2b and fluorouracil were 79.5%, 33.8% and 48.9%, respectively (Figure 3B, right). We also analyzed the cell cycle through propidium iodide (PI) staining. Specific S phase cell cycle inhibition was observed in Nova (23%) and IFN-α2b (18%) treatment groups (Figure 3C). The results showed that the anti-tumor activity of Nova, a protein originated from IFN-α, has been enhanced.

As shown in Figure 3C, besides the cell cycle change, our study demonstrates that the apoptosis rate of the Nova was 13.07%, while in rhIFN-α2b group, the apoptosis rate is 9.67%.

### Novaferon changes the expression of several apoptosis -related genes

**Figure 4:**
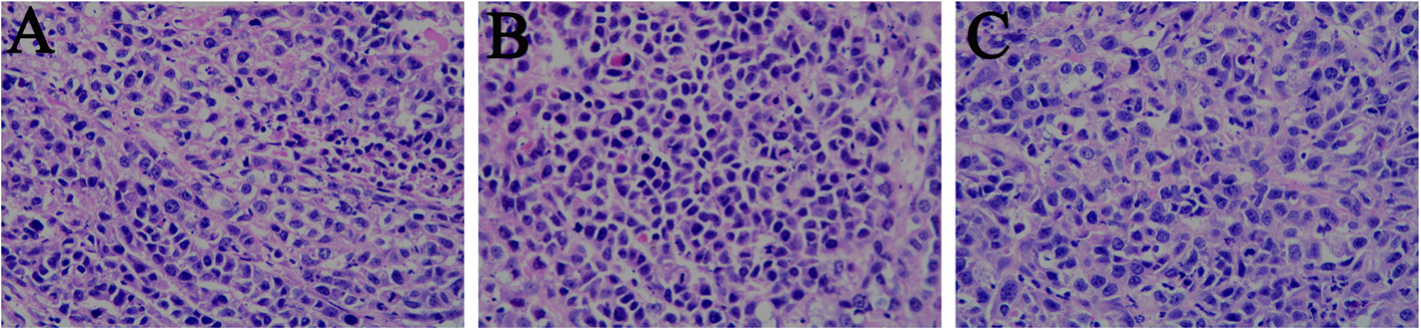
HE staining was observed under the microscope (200×), compared with negative control (A) we observed karyopyknosis and shape changed in the rhIFN- α 2B (B) and nova group (C).

HE staining was observed under the microscope, compared with negative control, we found that karyopyknosis and shape change of cells existed in the tumor sample treated with rhIFN- α 2B and nova (Figure 4).

**Figure 5:**
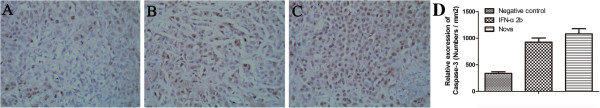
**The immuno histochemical analysis result of caspase 3 in each group.** Caspase-3 protein increased in dissected tumor tissues after mice was treated by IFN-α2b **(B)** and Nova **(C)** compared with the negative control **(A)**. **D** is quantization of the expression of the proteins evaluated in the different samples with immunohistochemistry.

**Figure 6:**
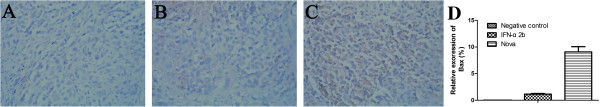
**The immuno histochemical analysis result of Bax in each group.** Bax protein increased in tumor tissues after mice was treated by IFN-α2b **(B)** and Nova **(C)** compared with the negative control **(A)**. **D** is quantization of the expression of the proteins evaluated in the different samples with immunohistochemistry.

**Figure 7:**
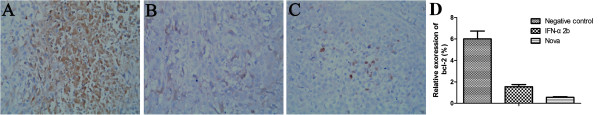
**The immuno histochemical analysis result of Bcl-2 in each group.** Bcl-2 protein decreased in tumor tissues after mice was treated by IFN-α2b **(B)** and Nova **(C)** compared with the negative control **(A)**. **D** is quantization of the expression of the proteins evaluated in the different samples with immunohistochemistry.

**Figure 8:**
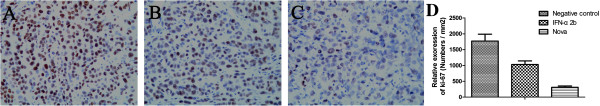
**Ki-67protein levels ingroup of IFN-α2b (B) and Nova (C) compared with the negative control (A) by immuno histochemical analysis. D** is quantization of the expression of the proteins evaluated in the different samples with immunohistochemistry.

**Figure 9:**
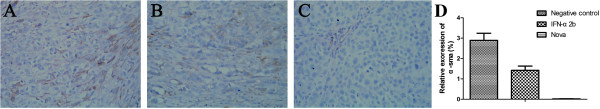
**α-SMA protein levels in groups of IFN-α2b (B) and Nova (C) compared with the negative control (A) by immuno histochemical analysis. D** is quantization of the expression of the proteins evaluated in the different samples with immunohistochemistry.

Caspases, Bax and Bcl-2 proteins were extensively studied for induction of apoptosis regulation. To explore the related apoptotic mechanism of Nova, we examined the protein levels of caspase-3, and Bcl-2 by immunohistochemistry analysis. Result showed caspase-3 protein increased in dissected tumor tissues after mice was treated by IFN-α2b and Nova compared with the negative control (Figure 5), the levels of caspase-3 was higher in Nova group than IFN-α2b group. The expression of Bax proteins (Figure 6) was consistent with caspase-3. On the other hand, Bcl-2 expression was down-regulated in IFN-α2b and Nova groups when compared with the control; the expression of Bcl-2 in Nova groups was less than IFN-α2b group (Figure 7). Moreover, expression of proliferation marker Ki-67 (Figure 8) and α-smooth muscle actin (α-SMA, Figure 9) were down-regulated in IFN-α2b and Nova groups.

### Binding of nova and IFN-α2b fused to IFN receptor 2

**Figure 10:**
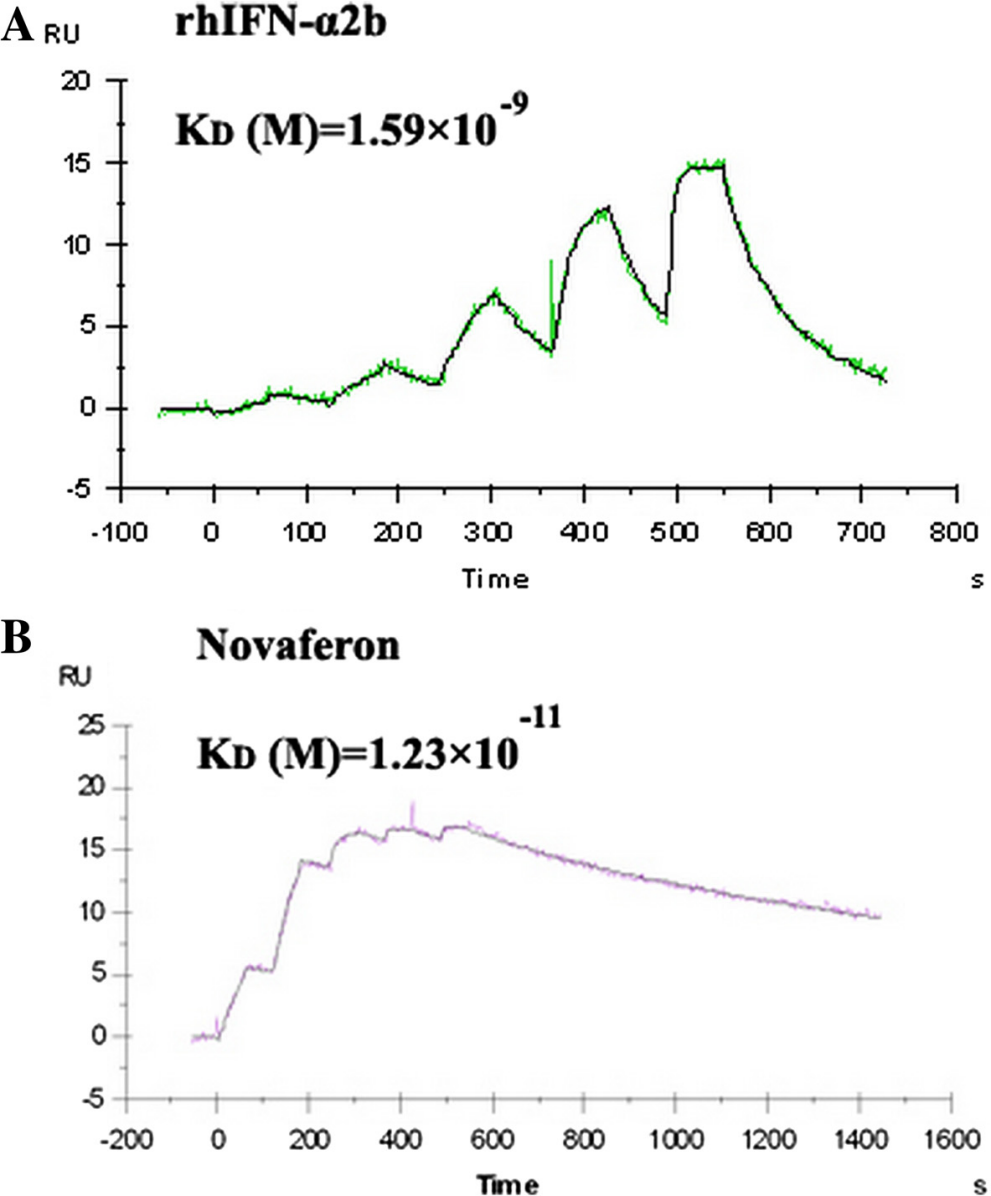
Binding curve showed that Nova (B) bind to IFNR2 with higher affinity than IFN-α2b (A).

SPR analysis showed Nova could interact with IFN receptor 2 (IFNR2) by Biacore T-100 instrument. Binding curve showed that Nova bind to IFNR2 with higher affinity (KD =1.23 × 10^-11^ M than that of rhIFN-α2b (Kd, 1.59 × 10^-9^ M) (Figure 10). This data demonstrates that IFNR2 is a probable receptor of Nova.

## Discussion

IFNs were the first cytokines used in treating malignant tumor, with the approval of recombinant human IFNs, IFN-α2a and IFN-α2b for the treatment of Hairy Cell Leukemia and Kaposi’s sarcoma [19], IFNs provide fundamental defense strategies against viral infections, tumor growth and immune disorders. We used rhIFN-α2b as a positive control to evaluate the therapeutic effect of Nova, results showed that Nova inhibited proliferation rate of the HepG2 cell in *vitro and in vivo* significantly. In our study, *in vivo* anti-cancer effect may include many mechanisms, such as immune regulation and cytokine regulation. During their cytotoxic effecting on tumor cells, IFN could activate monocytes and macrophages, thus produce free radicals (ROS) and reactive nitrogen intermediates (RNI) to exert their effect [20]. IFN can also cause decreased production of fibroblast growth factor (bFGF), and reduced transcription of vascular endothelial growth factor (VEGF) [6,21]. Therefore, *in vivo* tumor inhibition experiments exerted stronger inhibitory effects than *in vitro* tumor cell killing experiments. Moreover, Nova can interact with IFNR2 with a lower dissociation *in vitro*; therefore Nova could demonstrate higher biological activity than rhIFN-α2b.

Interferon is known to be involved in different phases of the mitotic cycle, the most common exerted effect is on the transition from G1 to S phase [22]. Type I and type II IFNs are capable of inhibiting the expression transcription factor c-myc, which is essential for activating cyclin-dependent kinase (CDK), subsequently regulate the transition from G1 to S phase and induce transcription of a large genes necessary for S phase [23]. The cell cycle analysis showed the percentage of S phase treated with Nova and rhIFN-α2b was 21.67% and 18.71% respectively, and the percentage of S phase of the blank control group was 14.75%. Compared with blank control group, Nova and IFN-a2b inhibit cell cycle progression by arresting them in S phase, so that the percentage of cells in S phase is increased.

Inhibition of DNA synthesis of S phase prevents mitotic entry through the action of the S-phase key point in tumor cells. Thus we speculate that one effect of Nova on these starved cells was to slow progression through S phase, and thus inhibited proliferation of the tumor.

Apoptosis, also known as programmed cell death, is a highly conserved eukaryotic cell suicide format [24]. Different from the anti-proliferate effect, apoptosis is removing aging cells through self-death or injury by the body and it is the genetic control of programmed cell death. Pro-apoptotic induction is one of the effective ways in anti-tumor therapy. Our study suggested that the ability of inducing apoptosis for Nova may be little stronger than rhIFN-α2b, but this result need to be further confirmed.

Cysteine-requiring Aspartate Protease (Caspase) is a family of proteases, it plays important role in the process of apoptosis. Caspase-3, belong to the caspase family CED-3 subfamily [25], is a key enzyme regulating apoptotic process. In addition, caspase-3 also plays a key role in chromatin condensation, DNA fragmentation during apoptosis. Caspase-3 pro-enzyme stays in the cytoplasm of normal cells, at the early stages of apoptosis, it is activated and induces apoptosis eventually [26]. Up-regulated Caspase-3 expression demonstrates that nova exerts its’ effect through inducing apoptosis in tumor cells [27].

Bax, also known as Bcl-2 associated protein X, is another member of the Bcl-2 family [28]. It is a pro-apoptotic protein in the mitochondria-dependent apoptosis pathway. In healthy mammalian cells, Bax is inactive in the cytoplasm. Once triggered by signals, Bax is transferred to the mitochondrial outer membrane, and begin to induce mitochondrial release of apoptosis gene factor, triggering the apoptotic response [29]. Interferon could up regulate Bak and Bax in response to apoptosis [30], which is consistent with our data. Also, we observed the down-regulation of anti-apoptotic proteins Bcl-2 in HepG2 tumor cells by Nova.

Ki-67, which is encoded by *MKI67*, is a cell proliferation-related nuclear protein used for diagnosing tumor cell proliferation [31]. Ki-67 expresses at the G1, S, G2 and mitosis phase, and is related with ribosomal RNA transcription [32]. Ki-67 staining is commonly used for diagnosis of malignant tumors. In our study; we observed that Ki67 in Nova treatment group is reduced than that in rhIFN-α2b group. The α-SAM was found in tumor vascular smooth muscle cells, is a common cancer-associated fibroblasts which has tumor protective role, in consistent with Ki-67, our study demonstrate Nova could down regulate expression of α-SAM and has better effect of anti-angiogenesis in tumor blood vessels than rhIFN-α2b.

Moreover, DNA contamination, endotoxin content, residual antibiotic activity and residual bacteria protein content of Nova is all below the limits of detection (data not shown). In a conclusion, Nova could be a potential powerful anti-tumor/virus drug; however, this also requires further clinical trials to verify.

## Competing interests

The authors declare there is no conflict of interest.

## Authors’ contributions

JZW defined the research theme. ML and CMR designed methods and experiments, carried out the laboratory experiments, analyzed the data, interpreted the results and wrote the paper. DNP, LW and YHL co-worked on associated data collection and their interpretation. KG and MRW co-designed experiments, discussed analyses, interpretation, and presentation. All authors read and approved the final manuscript.
